# Proteomic Analysis Suggests Altered Mitochondrial Metabolic Profile Associated With Diabetic Cardiomyopathy

**DOI:** 10.3389/fcvm.2022.791700

**Published:** 2022-03-02

**Authors:** Karina P. Gomes, Anshul S. Jadli, Luiz G. N. de Almeida, Noura N. Ballasy, Pariya Edalat, Ruchita Shandilya, Daniel Young, Darrell Belke, Jane Shearer, Antoine Dufour, Vaibhav B. Patel

**Affiliations:** ^1^Department of Physiology and Pharmacology, Cumming School of Medicine, Calgary, AB, Canada; ^2^Libin Cardiovascular Institute, Calgary, AB, Canada; ^3^McCaig Institute for Bone and Joint Health, Calgary, AB, Canada; ^4^Department of Cardiac Sciences, Cumming School of Medicine, Calgary, AB, Canada; ^5^Faculty of Kinesiology, University of Calgary, Calgary, AB, Canada; ^6^Department of Biochemistry and Molecular Biology, Cumming School of Medicine, University of Calgary, Calgary, AB, Canada

**Keywords:** diabetes, diabetic cardiomyopathy, diastolic dysfunction, electron transport chain, shotgun proteomics

## Abstract

Diabetic cardiomyopathy (DbCM) occurs independently of cardiovascular diseases or hypertension, leading to heart failure and increased risk for death in diabetic patients. To investigate the molecular mechanisms involved in DbCM, we performed a quantitative proteomic profiling analysis in the left ventricle (LV) of type 2 diabetic mice. Six-month-old C57BL/6J-lepr/lepr (*db/db*) mice exhibited DbCM associated with diastolic dysfunction and cardiac hypertrophy. Using quantitative shotgun proteomic analysis, we identified 53 differentially expressed proteins in the LVs of *db/db* mice, majorly associated with the regulation of energy metabolism. The subunits of ATP synthase that form the F1 domain, and Cytochrome c1, a catalytic core subunit of the complex III primarily responsible for electron transfer to Cytochrome c, were upregulated in diabetic LVs. Upregulation of these key proteins may represent an adaptive mechanism by diabetic heart, resulting in increased electron transfer and thereby enhancement of mitochondrial ATP production. Conversely, diabetic LVs also showed a decrease in peptide levels of NADH dehydrogenase 1β subcomplex subunit 11, a subunit of complex I that catalyzes the transfer of electrons to ubiquinone. Moreover, the atypical kinase COQ8A, an essential lipid-soluble electron transporter involved in the biosynthesis of ubiquinone, was also downregulated in diabetic LVs. Our study indicates that despite attempts by hearts from diabetic mice to augment mitochondrial ATP energetics, decreased levels of key components of the electron transport chain may contribute to impaired mitochondrial ATP production. Preserved basal mitochondrial respiration along with the markedly reduced maximal respiratory capacity in the LVs of *db/db* mice corroborate the association between altered mitochondrial metabolic profile and cardiac dysfunction in DbCM.

## Introduction

Diabetes mellitus (DM) is one of the major risk factors for cardiovascular disease (CVD), and CVD is the leading cause of morbidity and mortality worldwide. By 2045, DM is expected to affect 700 million people worldwide, with a prevalence of around 10.9% (1). In 1972, Rubler and colleagues first reported a *post-mortem* study of four diabetic patients who died of heart failure (HF) without evidence of hypertension, coronary artery disease, or congenital or valvular heart disease (2). This unique form of CVD was termed “diabetic cardiomyopathy” (DbCM). Since then, the pathophysiology of DbCM has been under investigation. However, its underlying molecular mechanisms have not yet been fully elucidated. Elusive molecular pathophysiology has resulted in the lack of standard treatment for DbCM.

The occurrence of DbCM is thought to be multifactorial, and various mechanisms have been proposed to be involved in diabetes-induced cardiac dysfunction, including resistance to metabolic actions of insulin, compensatory hyperinsulinemia, and progression of hyperglycemia in cardiac tissue (3). Together, these alterations result in changes in substrate metabolism and cardiac lipotoxicity (4), deposition of advanced glycated end-products (AGE) (5), endothelial and microvascular impairment (6), inappropriate neurohormonal responses (7), oxidative stress (8), subcellular component abnormalities, and maladaptive immune response (9). These changes result in myocardial injury, fibrosis, and hypertrophy leading to diastolic, and eventually systolic, heart failure (10).

The prevalence of DbCM has been estimated between 30% and 60% in preclinical and clinical stages among the diabetic population (11). Although significant progress has been made in recent years in the diagnosis and management of DbCM, until now, there is no specific therapy for myocardial damage induced by DbCM. A better understanding of the underlying pathological mechanisms of DbCM is highly warranted to further improve the clinical management, and therapy, of DbCM. To decipher the underlying mechanisms involved in DbCM at the molecular level, advanced proteomic profiling of left ventricular (LV) tissue specimens from type 2 diabetic mice was carried out.

## Materials and Methods

### Experimental Animals

All experiments were performed in accordance with the University of Calgary institutional guidelines, which conform to guidelines published by the Canadian Council on Animal Care and the Guide for the Care and Use of Laboratory Animals published by the U.S. National Institutes of Health (revised 2011). Animals were kept at the animal facilities of the Health Sciences Animal Resources Centre of the University of Calgary. Six-month old male C57BL/6J-lepr/lepr (*db/db*) and age-matched C57BL/6J (wild-type [WT]) mice were used as experimental units, and a total of 37 mice were used in the current study. Mice were housed in standard animal cages and maintained in a constant environment with controlled room temperature, humidity, and light-dark cycle. They had access to laboratory chow pellets and drinking water *ad libitum* throughout the study, except before the oral glucose tolerance test, when the animals were fasted for 6 h before the procedure. All studies were approved by the Animal Care Committee of the University of Calgary.

### Oral Glucose Tolerance Test

An oral glucose tolerance test was performed in 6 h fasted conscious mice, as previously described (12). Briefly, mice were administered with glucose (1 g/kg) by oral gavage, and the blood glucose levels were monitored repeatedly at 0, 15, 30, 60, 90, and 120 min post-glucose administration. Blood glucose levels were plotted against the time curve to determine glucose tolerance.

### Echocardiography and Tissue Doppler Imaging

Cardiac function was evaluated using the Vevo 3100 high-resolution imaging system equipped with a 30-MHz transducer (MX250, VisualSonics) (12–14). Mice were anesthetized with 1.5% isoflurane in 100% oxygen and kept on a heating pad, with body temperature maintained at 36.5–37.5°C. Pre-warmed ultrasound gel was placed on the shaved chest of the anesthetized mouse. The temperature and heart rate were constantly monitored during the scanning. M-mode echocardiography images were obtained to measure LV anterior and posterior wall thickness, and LV end-diastolic and end-systolic dimensions, which were used to calculate fractional shortening (FS) and ejection fraction (EF), measures of the LV systolic function. Diastolic transmitral LV inflow images were obtained from apical four-chamber views using color flow mapping-guided pulsed-wave Doppler and were used to measure early (E) and late (atrial, A) peak filling blood flow velocities (and calculate E/A ratio), isovolumic relaxation time (IVRT), and deceleration time, all commonly used indices of LV diastolic function). Transmitral flow and tissue Doppler imaging were used to assess the E/E' ratio. All echocardiographic images were analyzed using Vevo LAB ultrasound analysis software (v5.5.1).

### Shotgun Proteomic Analysis

Mice were euthanized under ketamine and xylazine anesthesia. The hearts were immediately dissected, and the LVs were stored in a −80°C for proteomics analysis. Subsequently, protein samples were lysed with 1% sodium dodecyl sulfalte (SDS), 0.1 M dithiothreitol (DTT) in 200 nM HEPES (pH 8), protease inhibitor tablets (Sigma Aldrich, ON, Canada) with a final concentration of 3 M guanidine HCl (pH 8), 100 mM HEPES, and 10 mM DTT. Samples were alkylated by incubation with a final concentration of 15 mM iodoacetamide (IAA) in the dark for 25 min at room temperature, and the pH was adjusted to 6. Samples were then trypsinized overnight at 37°C using Trypsin gold (Promega, WI, USA). The next day, samples were incubated for 18 h at 37°C with isotopically heavy [40 mM 13CD_2_O + 20 mM NaBH_3_CN (sodium cyanoborohydride)] or light labels [40mM light formaldehyde (CH_2_O) + 20 mM NaBH_3_CN], to label peptide α- and ε-amines. Samples were passed through a C18 chromatography before being subjected to liquid chromatography and tandem mass spectrometry (LC-MS/MS).

### High-Performance Liquid Chromatography and Mass Spectrometry

Liquid chromatography and mass spectrometry experiments were performed at the Southern Alberta Mass Spectrometry (SAMS) core facility at the University of Calgary, Canada. An Orbitrap Fusion Lumos Tribrid mass spectrometer (Thermo Scientific) operated with Xcalibur (version 4.0.21.10) and coupled to a Thermo Scientific Easy-nLC (nanoflow Liquid Chromatography) 1200 system was used for the analysis. Tryptic peptides (2 μg) were loaded into a C18 trap (75 um x 2 cm; Acclaim PepMap 100, P/N 164946; ThermoScientific) at a flow rate of 2 μl/min of solvent A (0.1% formic acid and 3% acetonitrile in LC-MS grade water). Peptides were eluted using a 120 min gradient from 5 to 40% (5 to 28% in 105 min followed by an increase to 40% B in 15 min) of solvent B (0.1% formic acid in 80% LC-MS grade acetonitrile) at a flow rate of 0.3 μL/min and separated on a C18 analytical column (75 um × 50 cm; PepMap RSLC C18; P/N ES803; Thermo Scientific).

Peptides were subsequently electrosprayed using a voltage of 2.3 kV into the ion transfer tube (300°C) of the Orbitrap Lumos operating in positive mode. Orbitrap first performed a full MS scan at a resolution of 120,000 FWHM to detect the precursor ion with an m/z between 375 and 1,575 and a +2 to +7 charge. The Orbitrap Auto Gain Control (AGC) and the maximum injection time were set at 4 × 10^5^ and 50 ms, respectively. Orbitrap was operated using full speed mode with a 3 sec cycle time for precursor selection. The most intense precursor ions showing a peptidic isotopic profile and having an intensity threshold of at least 5,000 were isolated using the quadrupole and fragmented with HCD (30% collision energy) in the ion routing multipole. Fragment ions (MS2) were analyzed in the ion trap at a fast scan rate. The AGC and the maximum injection time were set at 1 x 10^4^ and 35 ms, respectively, for the ion trap. Dynamic deletion was enabled for 45 sec to avoid acquiring the same precursor ion with a similar m/z (plus or minus 10 ppm).

### Proteomic Data Analysis

Spectral matching of the resulting raw data was done in MaxQuant (15) software package (v.1.6.10.23) implemented with the Andromeda algorithm using a UniProt murine proteome database, at a peptide-spectrum match false discovery rate of <0.01. Search parameters included a mass tolerance of 20 p.p.m. for the parent ion, 0.5 Da for the fragment ion, carbamidomethylation of cysteine residues (+57.021464 Da), variable N-terminal modification by acetylation (+42.010565 Da), and variable methionine oxidation (+15.994915 Da). N-terminal and lysine heavy (+34.063116 Da) and light (+28.031300 Da) dimethylation were defined as labels for relative quantification. The cleavage site specificity was set to Trypsin/P for the proteomics data, with up to two missed cleavages allowed. Significant outlier cut-off values were determined after Log2 transformation by boxplot-and-whiskers analysis using the BoxPlotR tool (16). The dataset was deposited into the PRIDE database and is freely available using the accession code PXD029566.

### Protein–Protein Interactions and Pathway Analysis Using Bioinformatics

The selected proteins were uploaded to Metascape (17) to validate various biological functions of the selected proteins. The Search Tool for the Retrieval of Interacting Genes (STRING) database was used to identify interconnectivity among proteins. The protein interaction relationship is encoded into networks in the STRING v11 database (https://string-db.org). *Mus musculus* was used as our model organism at a false discovery rate of 1%.

To further appreciate their biological significance, the differentially expressed proteins were subjected to protein network analysis using the Ingenuity Pathway Analysis (IPA) software (Qiagen Inc.) based on curated databases from the literature. These include binding, activation, inhibition, expression, and other protein interactions to generate pathways according to the function of the molecules involved. IPA is a powerful tool widely used in the omics field to suggest/predict the effects of specific conditions or drugs on biological outcomes. Datasets containing protein identifiers (UniProt) and corresponding expression values (Log2 [Fold change]) of *db/db* vs. age-matched control were uploaded, and predicted networks were analyzed.

### Mitochondrial Bioenergetics

Bioenergetics profile of isolated mitochondria was assessed using Seahorse Analyzer XFe24 (Agilent technologies) (18, 19). Briefly, mitochondria were isolated from the LVs of *db/db* and age-matched WT mice using Dounce homogenization and differential centrifugation as directed by mitochondrial isolation kit for tissue (#ab110168, Abcam). Isolated mitochondria were resuspended in mitochondrial assay solution [MAS; 220 mM mannitol, 10 mM KH2PO4, 5 mM MgCl2, 2 mM HEPES, 1 mM EGTA, and 0.2% (w/v) fatty acid-free BSA, pH 7.2] containing 10 mM of Glutamate (#G8415, Sigma-Aldrich) and 5 mM Malate (#M6413, Sigma-Aldrich) for complex I-driven respiration. The total protein concentration of isolated mitochondria was determined by a BCA assay (#5000116, Bio-Rad). A stock solution of 40 mM ADP (#A2754, Sigma-Aldrich) was prepared in MAS. Stocks of inhibitors and uncouplers were prepared by dissolving 10 mM FCCP (#C2920, Sigma), 5 mg/mL of oligomycin (#O4876, Sigma), and 40 mM of antimycin A (#A8674, Sigma) in DMSO. The mitochondrial coupling assay for isolated mitochondria using substrates specific for the respiratory chain complex I (RCCI) was performed as described previously (18–20). 50 μl suspension of 10 μg isolated mitochondria was loaded in each well of XFe24 plates, except the wells intended for the background correction. Following final concentrations of substrate, inhibitors and uncouplers were used in wells for RCCI-driven respiration: 4 nM ADP (Port A), 2.5 μg/ml oligomycin (Port B), 4 μM FCCP (Port C), and 4 μM Antimycin A (Port D). All data were analyzed using the XFe Wave software (version 2.6; Agilent Technologies) and displayed as point-to-point oxygen consumption rates (pmol/min/well). Data are presented as the average of 3 replicate wells ±SEM.

### Western Blot

Western blot analysis was performed in the mitochondrial and cytoplasmic fractions obtained by the mitochondrial isolation kit for tissue (#ab110168, Abcam) as well as in the whole tissue lysates from LVs of *db/db* and WT mice to validate the purity of the mitochondrial isolation (**Figure 7F**). Briefly, equal amounts of proteins were separated using SDS-PAGE and electrophoretically transferred to PVDF membranes. Non-specific binding was blocked by incubation in 5% non-fat milk and 0.1% Tween 20 in Tris-buffered saline. The membranes were probed individually with specific primary antibodies against glyceraldehyde 3-phosphate dehydrogenase (GAPDH; 1:1000, #sc-32233, Santa Cruz Biotechnology), voltage-dependent anion channel 1 (VDAC1; 1:1000, #sc-390996, Santa Cruz Biotechnology) and NADH dehydrogenase (ubiquinone) 1 β subcomplex subunit 11 (NDUFB11; 1:1000, #sc-374370, Santa Cruz Biotechnology). After probing with the HRP-linked secondary antibody (anti-mouse IgG, 1:3000, #7076, Cell Signaling Technology), membranes were incubated with SuperSignal West Femto Maximum Sensitivity Substrate (#34096; Thermo Scientific), and chemiluminescence was recorded using iBright™ FL1500 Imaging System (Invitrogen). Immunoreactive bands were quantified by the iBright Analysis Software using the total protein detection (No-Stain Protein Labeling Reagent, #A44717, Invitrogen) as a normalization control.

### Statistical Analysis

All data are presented as mean±SEM. The sample sizes were determined based on 95% confidence level. Hypothesis testing methods included unpaired and two-tailed Student's *t*-test (two independent groups) and repeated measures ANOVA followed by Sidak's multiple comparisons. Statistical comparisons were performed by GraphPad Prism software. Statistical significance is recognized at *p* < 0.05.

## Results

### *db/db* Mice Exhibit Diastolic Dysfunction and Cardiac Remodeling

**Figure 1:**
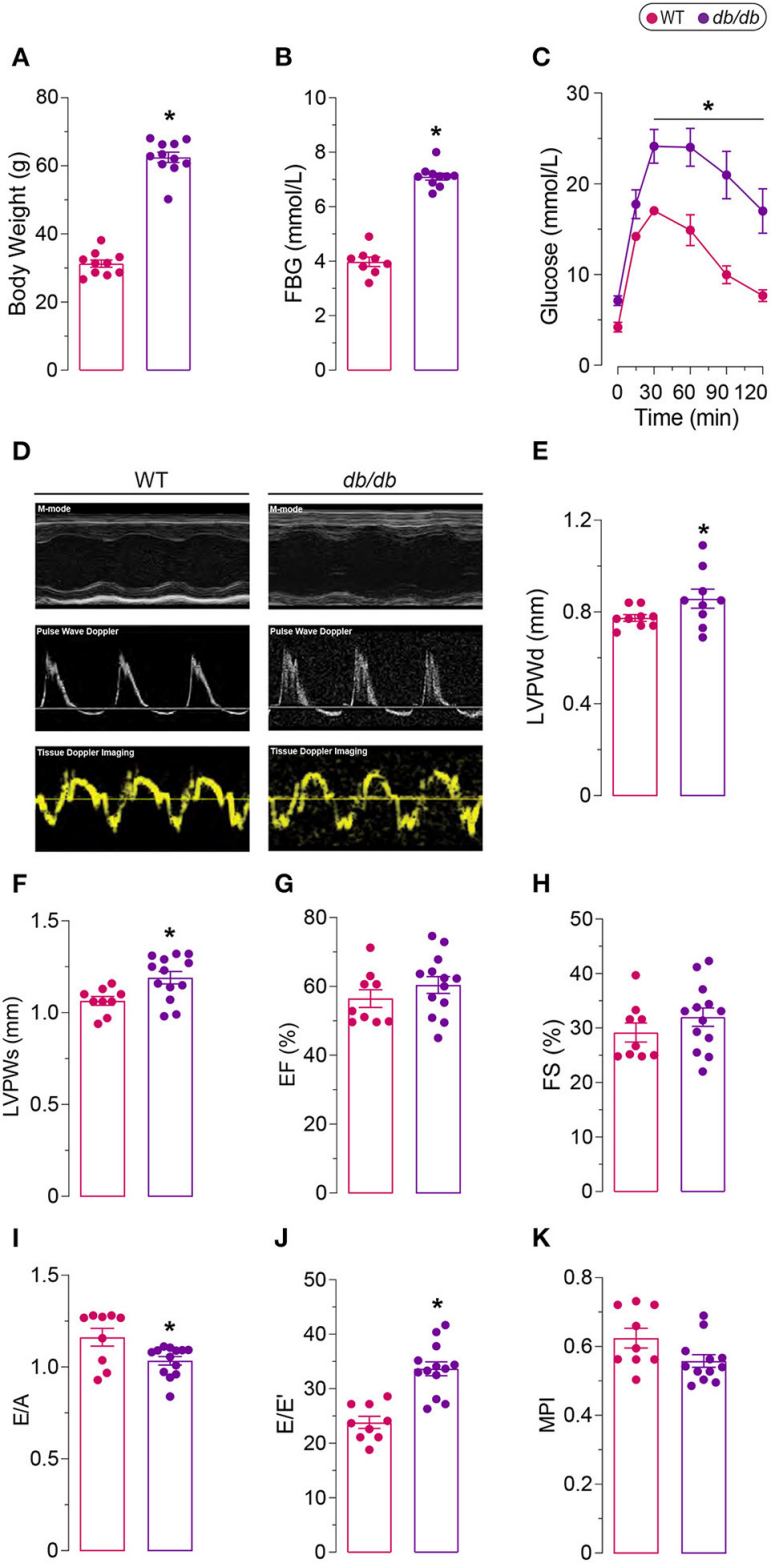
Diabetic phenotype of 6 months old *db/db* mice. **(A)** Increased body weight, **(B)** fasting blood glucose (FBG), and **(C)** glucose intolerance in *db/db* mice compared to age-matched controls (WT). **(D)** Representative M-mode, Pulse Wave Doppler, and Tissue Doppler Imaging echocardiography. Increased left ventricle posterior wall thickness in **(E)** diastole (LVPWd) and in **(F)** systole (LVPWs) indicate left ventricular hypertrophy. **(G)** Ejection fraction (EF) and **(H)** fractional shortening (FS) remained unaffected, indicating preserved systolic function in *db/db* mice. **(I)** Decreased ratio of early to late filling (E/A) and **(J)** increased ratio of late filling in Pulse Wave Doppler to the late filling in Tissue Doppler (E/E') show abnormal diastolic function. **(K)** Myocardial performance index (MPI) was not different between the groups. *Represents *p*-value <0.05 between WT (*n* = 8–10) and *db/db* (*n* = 9–13) mice. The scale bar shows 1.5 mm [y-axis of **(D)**], 100 ms [x-axis of **(D)**].

Leptin-receptor mutant *db/db* mice exhibit many of the clinical characteristics of type 2 diabetes and metabolic syndrome, including hyperglycemia, hyperinsulinemia, obesity, hypertension, hyperlipidemia, and glucose intolerance (21, 22). We observed significantly increased obesity in *db/db* mice at 6 months of age (Figure 1A). Increased obesity in *db/db* mice was also associated with increased fasting blood glucose levels and glucose intolerance, which validated the induction of severe type 2 diabetes in 6 months old *db/db* mice (Figures 1B,C). Chronic type 2 diabetes in 6 months old *db/db* mice resulted in the onset of DbCM (Table 1; Figures 1D–K). Quantitative assessments of transthoracic echocardiography and tissue Doppler imaging are presented in Table 1. Compared with age-matched WT mice, *db/db* mice exhibited significantly increased LV Posterior Wall thickness at diastole (LVPWd) (Figures 1D,E) and systole (LVPWs) (Figures 1D,F), indicative of cardiac hypertrophy in *db/db* mice. However, no significant differences were observed in systolic function among diabetic and non-diabetic mice (Figures 1D,G,H; Table 1). *db/db* mice showed reduced E/A ratio (Figure 1I), and markedly increased E/E' ratio, a sensitive indicator of diastolic dysfunction (Figure 1J). However, myocardial Performance Index (MPI), an index that incorporates both systolic and diastolic time intervals in expressing global systolic and diastolic ventricular function was not different between the groups (Figure 1K). Echocardiographic phenotyping validated the onset of DbCM characterized by diastolic dysfunction and cardiac hypertrophy, without any overt systolic dysfunction in 6 months old male *db/db* mice.

**Table 1:** Body weight and echocardiographic parameters of 6-wk-old control (WT) and diabetic *(db/db)* mice.

	**WT**	***db/db***	***p***
	***(n* = 13)**	**(*n* = 9)**	
HR, beats/min	428.6 ± 6.50	407.1 ± 5.05^*^	0.0157
LVPWd, mm	0.774 ± 0.0145	0.857 ± 0.0416^*^	0.0474
LVPWs, mm	1.064 ± 0.0236	1.191 ± 0.0333^*^	0.0106
LVIDd, mm	3.733 ± 0.1167	3.754 ± 0.0500	0.8643
LVIDs, mm	2.678 ± 0.1299	2.585 ± 0.0939	0.5574
IVSd, mm	0.877 ± 0.0364	0.915 ± 0.0421	0.5323
IVSs, mm	1.078 ± 0.0400	1.146 ± 0.0501	0.3342
EF, %	56.48 ± 2.568	60.42 ± 2.440	0.2917
FS, %	29.19 ± 1.737	32.01 ± 1.690	0.2722
Vcf, circs/s	0.611 ± 0.0388	0.608 ± 0.0287	0.9537
ET, ms	49.44 ± 0.9308	51.11 ± 0.9311	0.0745
IVCT, ms	14.29 ± 0.8149	13.02 ± 0.346	0.1229
IVRT, ms	15.16 ± 0.8235	15.10 ± 0.4649	0.9503
E/A	1.162 ± 0.0486	1.034 ± 0.0230^*^	0.0160
MPI	0.6244 ± 0.0284	0.5579 ± 0.0182	0.0538

*Values are mean ± SEM. HR, heart rate; LVPWd, left ventricular posterior wall thickness in diastole; LVPWs, left ventricular posterior wall thickness in systole; LVIDd, diastolic left ventricular (LV) internal dimension; LVIDs, systolic LV internal dimension; IVSd, Interventricular septal end diastole; IVSs, interventricular septal end systole; EF, ejection fraction; FS, fractional shortening; Vcf, velocity of circumferential shortening; ET, ejection time; IVCT, isovolumic contraction time; IVRT, Isovolumic relaxation time; E/A, early rapid filling/atrial contraction; MPI, myocardial performance index.*

* *P < 0.05 compared with age-matched controls*.

### Differential Protein Expression in the LV of *db/db* Mice

**Figure 2:**
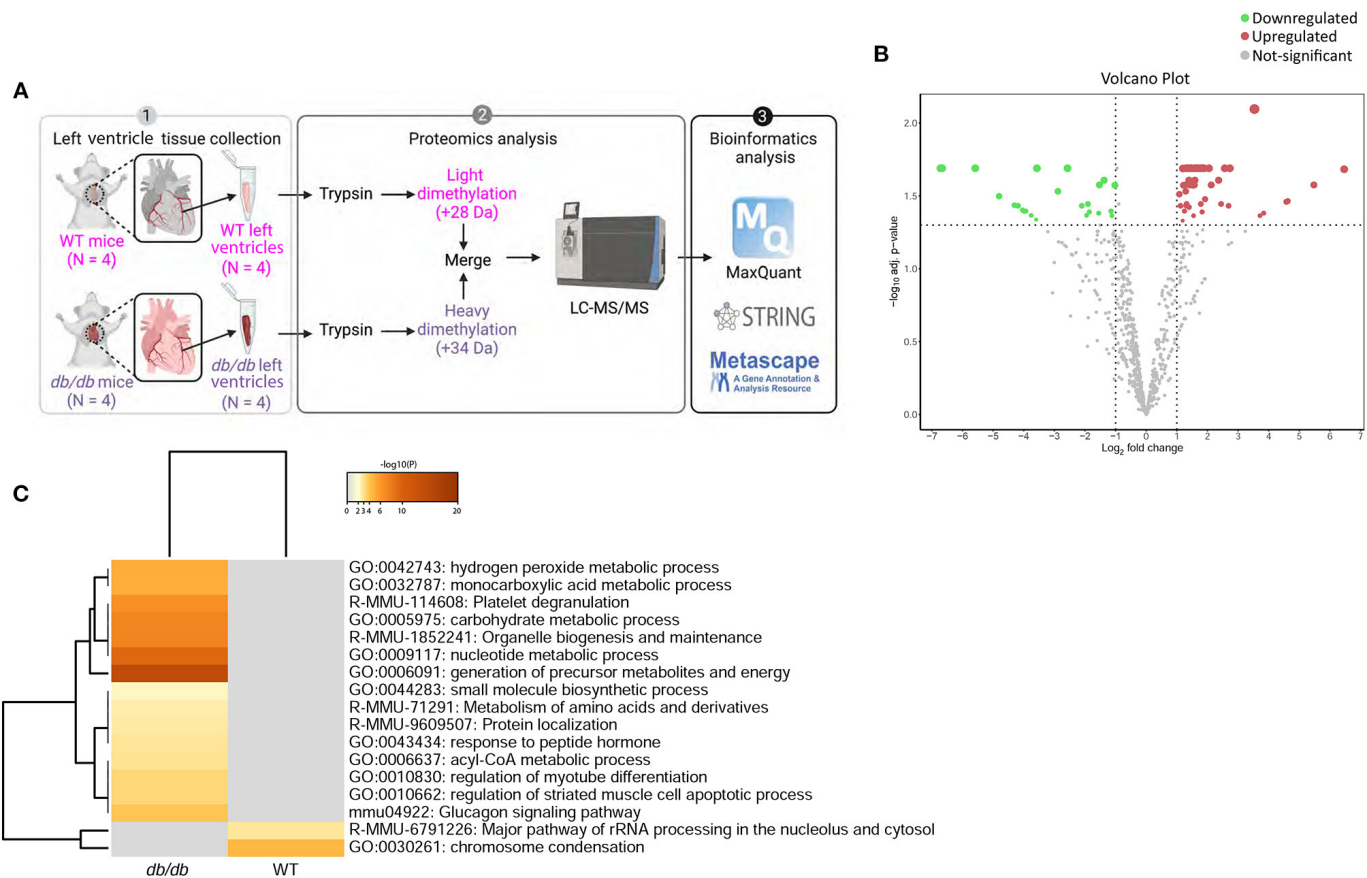
Process enrichment analysis of differentially expressed proteins in *db/db* mice. **(A)** Experimental design. Left ventricles from WT-control (n = 4) and *db/db* (n = 4) mice were labeled with light or heavy formaldehyde, quantified by shotgun proteomics and analyzed using MaxQuant. **(B)** Volcano plot of all proteins identified. The points indicate different proteins that display both large magnitude fold-changes (x-axis) and high statistical significance (y-axis). The dashed horizontal line shows the *p*-values cut-off, and the two vertical dashed lines indicate downregulated (green) and upregulated (red) proteins in the left ventricle of *db/db* mice. Gray points show the non-significantly differently expressed proteins. **(C)** Heatmap of top 17 enriched terms across upregulated and downregulated proteins in *db/db* mice using Metascape. Darker color indicates a lower *p*-value.

Quantitative shotgun proteomics analysis performed after light (+28 Da) and heavy (+34 Da) formaldehyde labeling (demethylation) resulted in the identification of 715 proteins, which were subsequently used for comparative analysis (Figure 2A). As shown on the volcano map (Figure 2B), on the basis of an absolute fold change in expression levels and a corrected *p*-value (*p* < 0.05), we found 53 proteins that were differentially expressed in *db/db* LVs compared to the WT LVs. Among the 53 differentially expressed proteins in LV of *db/db* mice, 30 proteins were downregulated in response to chronic diabetes, while 23 were upregulated. All the differentially expressed proteins are listed in Table 2. In addition to the numerous peptides differently expressed in *db/db* mice revealed by the proteomic profile, the Metascape (17) analysis identified a top enrichment in the generation of precursor metabolites and energy production (GO:0006091) (Figure 2C).

**Table 2:** Selected peptides that were differentially regulated in the left ventricle of *db/db* mice.

**Gene**	**Protein name**	**Log2 (db/db:WT)**	**Adj. *p* Value**	
S100a1	Protein S100-A1	6.4633	0.0207	U P R E G U L A T E D
Rpl17	60S ribosomal protein L17	5.4742	0.0266		
Ehd1;Ehd3;Ehd4	EH domain-containing protein	4.6209	0.0343		
Tmx1	Thioredoxin-related transmembrane protein 1	4.5741	0.0347		
Zscan4b;Zscan4c;Zsc an4f;Zscan4d	Zinc finger and SCAN domain-containing	3.8345	0.0415		
Hp	Haptoglobin	3.5337	0.0080		
B4galt1	Beta-1,4-galactosyltransferase 1	3.2367	0.0553		
Fam122a	P2R1A-PPP2R2A-interacting phosphatase regulator 1	2.7861	0.0661		
Gm20390;Nme2	Nucleoside diphosphate kinase B	2.7300	0.0204		
Nfs1;Gm28036	Cysteine desulfurase, mitochondrial	2.6907	0.0682		
Acta1;Actc1;Acta2	Actin, alpha skeletal muscle	2.6882	0.0370		
Ighg2b;Igh-3	Ig gamma-2B chain C region	2.6631	0.0553		
Atp5d	ATP synthase subunit delta	2.5640	0.0204		
Znfx1	NFX1-type zinc finger-containing protein 1	2.4443	0.0359		
Gm3839;Gapdh;Gapd hs	Glyceraldehyde-3-phosphate dehydrogenase	2.3627	0.0247		
2210016F16Rik	Queuosine salvage protein	2.1222	0.0266		
Adipoq	Adiponectin	2.0487	0.0204		
Igkv1-110;Igkv1- 35;Igkv1-99;Igkv1-115	Immunoglobulin kappa variable	1.9245	0.1173		
Gm20425;Tf;Trf	Telomeric repeat-binding factor 1	1.9215	0.0333		
Psma3	Proteasome subunit alpha type-3	1.9042	0.1174		
Atp5i	ATP synthase subunit e, mitochondrial	1.8973	0.1174		
Gys1	Glycogen [starch] synthase, muscle	1.8882	0.0204		
Atp5b	ATP synthase subunit beta, mitochondrial	1.7560	0.0204		
Cyc1	Cytochrome c1, heme protein, mitochondrial	1.5430	0.0432		
Atp5a1	ATP synthase subunit alpha, mitochondrial	1.4200	0.0265	
Ndufb11	NADH dehydrogenase [ubiquinone] 1 beta subcomplex subunit 11, mitochondrial	−1.1490	0.0402	D O W N R E G U L A T E D
Adck3	Atypical kinase COQ8A, mitochondrial	−1.3760	0.0246		
Gorasp2	Golgi reassembly-stacking protein 2	−2.1490	0.0977		
Pxn	Paxillin	−2.1530	0.1426		
Myh7;Myh6;Myh4;Myh 3;Myh1;Myh2;Myh8;M yh7b	Myosin	−2.1923	0.0948		
Rps4x;Gm15013	40S ribosomal protein S4, X isoform	−2.2322	0.0626		
Nedd8	NEDD8	−2.2950	0.0775		
Apoa1	Apolipoprotein A-I	−2.4082	0.0805		
Prosc	Pyridoxal phosphate-binding protein	−2.4441	0.0795		
Idh3a	Isocitrate dehydrogenase [NAD] subunit alpha, mitochondrial	−2.4736	0.2108		
C5	Complement C5	−2.5709	0.0204		
Cand1	Cullin-associated NEDD8-dissociated protein 1	−2.6097	0.0705		
Aldoa;Aldoc	Fructose-bisphosphate aldolase	−2.6662	0.0689		
Cmya5	Cardiomyopathy-associated protein 5	−2.7514	0.0661		
Mrps24	28S ribosomal protein S24, mitochondrial	−2.7644	0.0661		
Pabpc4;Gm10110	Polyadenylate-binding protein	−2.8658	0.0653		
Tpx2	Targeting protein for Xklp2	−2.8850	0.0294		
Idh1	Isocitrate dehydrogenase [NADP] cytoplasmic	−3.0045	0.0905		
Cfl1	Cofilin-1	−3.0594	0.1464		
Nrbp1	Nuclear receptor-binding protein	−3.2125	0.0556		
Fnbp1	Formin-binding protein 1	−3.5705	0.0204		
Map2	Microtubule-associated protein 2	−3.5947	0.0460		
Rps6ka5	Ribosomal protein S6 kinase alpha-5	−3.7585	0.0430	
Atp2a1;Atp2a3	Sarcoplasmic/endoplasmic reticulum calcium ATPase 1	−3.9354	0.0402	
Zfp280d;Znf280d	Zinc finger protein 280D	−3.9930	0.0402	
Smc2	Structural maintenance of chromosomes protein 2	−4.0378	0.0395	
Myo3a	Myosin-IIIa	−4.1878	0.0370	
Pclo	Protein piccolo	−4.3033	0.0366	
Noxred1	NADP-dependent oxidoreductase domain-containing protein 1	−4.8053	0.0318	
Rdm1	RAD52 motif-containing protein 1	−5.5836	0.0204	
Cblb	E3 ubiquitin-protein ligase CBL-B	−6.6663	0.0204	
Cep162	Centrosomal protein of 162 kDa	−6.7192	0.0204	

*Color gradient represents the magnitude of the changes. Shades of red refer to upregulation and shades of green refer to downregulation*.

**Figure 3:**
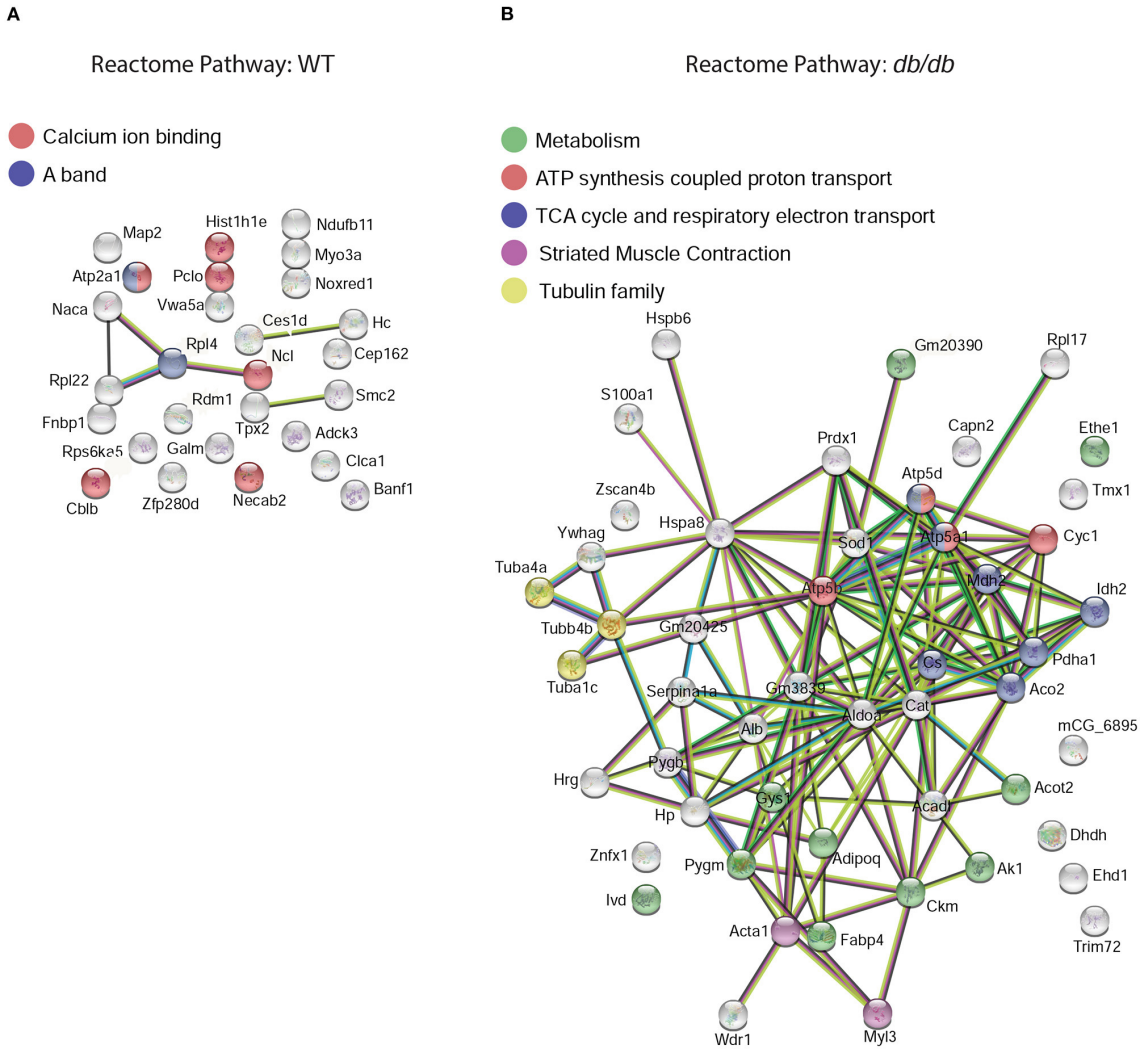
Functional protein-protein network analysis by STRING database. Left ventricular elevated proteins in **(A)** WT or **(B)** *db/db* mice identified from the proteomic analysis were mapped by searching the STRING software with a confidence level of 1% false discovery rate. The global protein network shows the shift in reactome priorities from calcium handling and muscle contraction in healthy mice, to energy metabolism and mitochondrial dysfunction in DbCM. Colored lines between the proteins indicate different types of interaction evidence: known interactions (teal), experimentally determined (pink), predicted interactions gene neighborhood (green), gene fusions (red), gene co-occurrence (blue), text-mining (yellow), co-expression (black), protein homology (purple).

To characterize the changes in the global protein network in DbCM, we investigated the functional interactions of altered proteins for each condition using STRING (23) protein-protein interaction networks and functional enrichment analysis. In the LV of WT mice, we identified two dominant clusters: six enriched proteins were involved in calcium ion binding, and two were involved in the striated muscle contraction (Figure 3A). However, in the LV of *db/db* mice we found enrichment for nine proteins involved in metabolism, seven involved in the citric acid cycle and respiratory electron transport, four involved in ATP synthesis-coupled proton transport, three of the tubulin family, and two involved in striated muscle contraction (Figure 3B).

### Ingenuity Pathway Analysis Suggests Mitochondrial Dysfunction in *db/db* Mice Hearts

**Figure 4:**
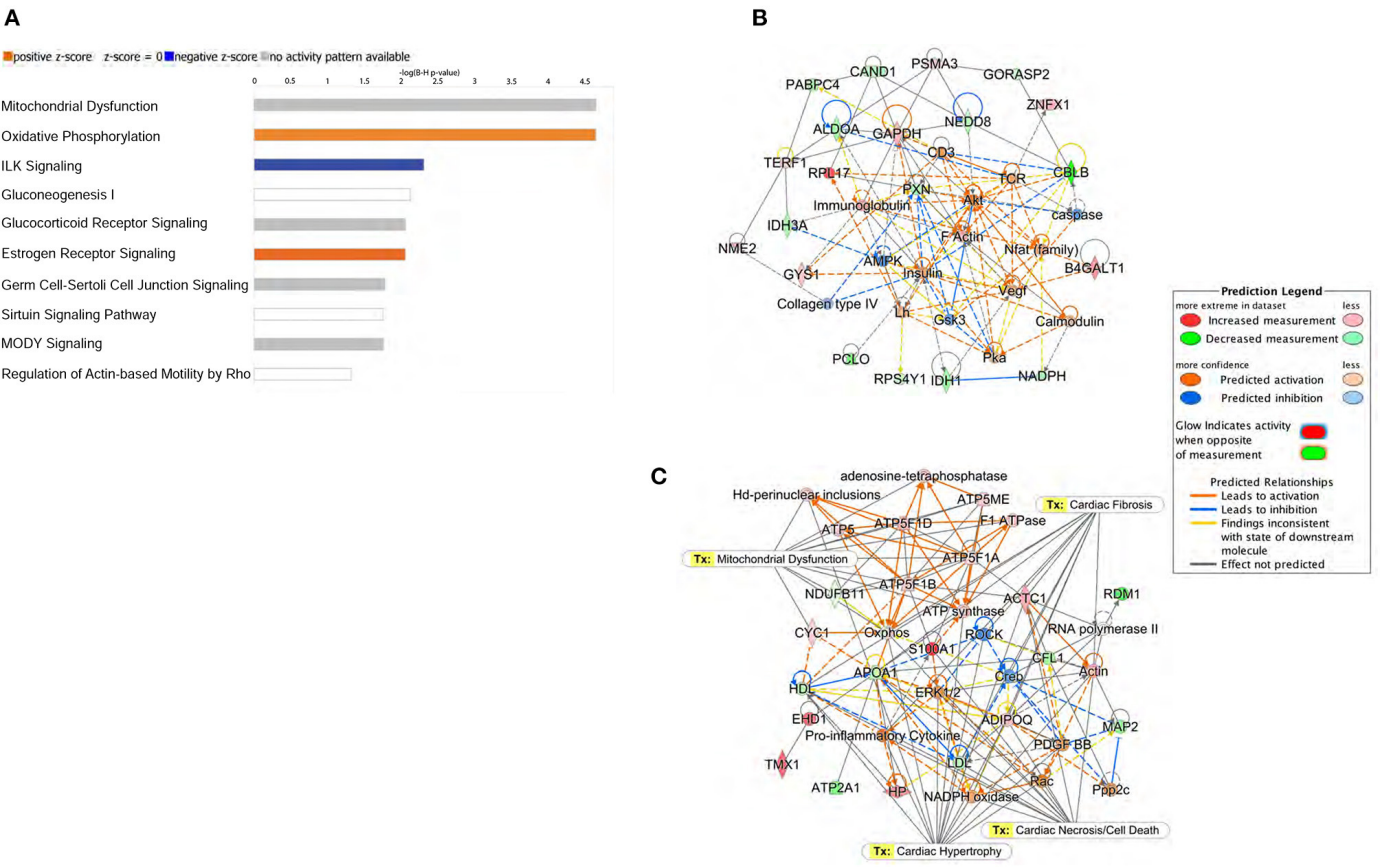
Ingenuity Pathway Analysis (IPA) of proteomic data. **(A)** Top 10 canonical pathways identified by IPA of proteins differentially expressed in left ventricle of *db/db* mice. The most statistically significant canonical pathways identified in *db/db* mice are listed according to their *p*-value (–log). Blue bars: negative z-score; orange bars: positive z-score. Clear bars are indicative of a z-score of 0, and thus have no difference in activity. Gray shaded bars indicate that there is no activity pattern available identified in IPA, despite highly significant association of the proteins within the pathway. Predictive bioinformatics analysis revealed that the significantly changed proteins participated in various biological processes. **(B)** Carbohydrate metabolism, **(C)** energy production, and cellular assembly were the highest scoring molecular networks identified by IPA in *db/db* mice.

Specific canonical pathways and their networks functions were further explored using the IPA bioinformatics program to compare common-specific proteins and their pathological or functional implications. Canonical pathway analysis through IPA identified the top 10 pathways in *db/db* mice as described in Figure 4A. Predictive bioinformatics analysis revealed that the differentially expressed proteins participated in various biological processes, such as carbohydrate metabolism (Figure 4B), mitochondrial dysfunction, cardiac hypertrophy, cardiac necrosis/cell death, and cardiac fibrosis (Figure 4C), the biological responses that are previously known to occur in diabetic hearts (24–26).

**Figure 5:**
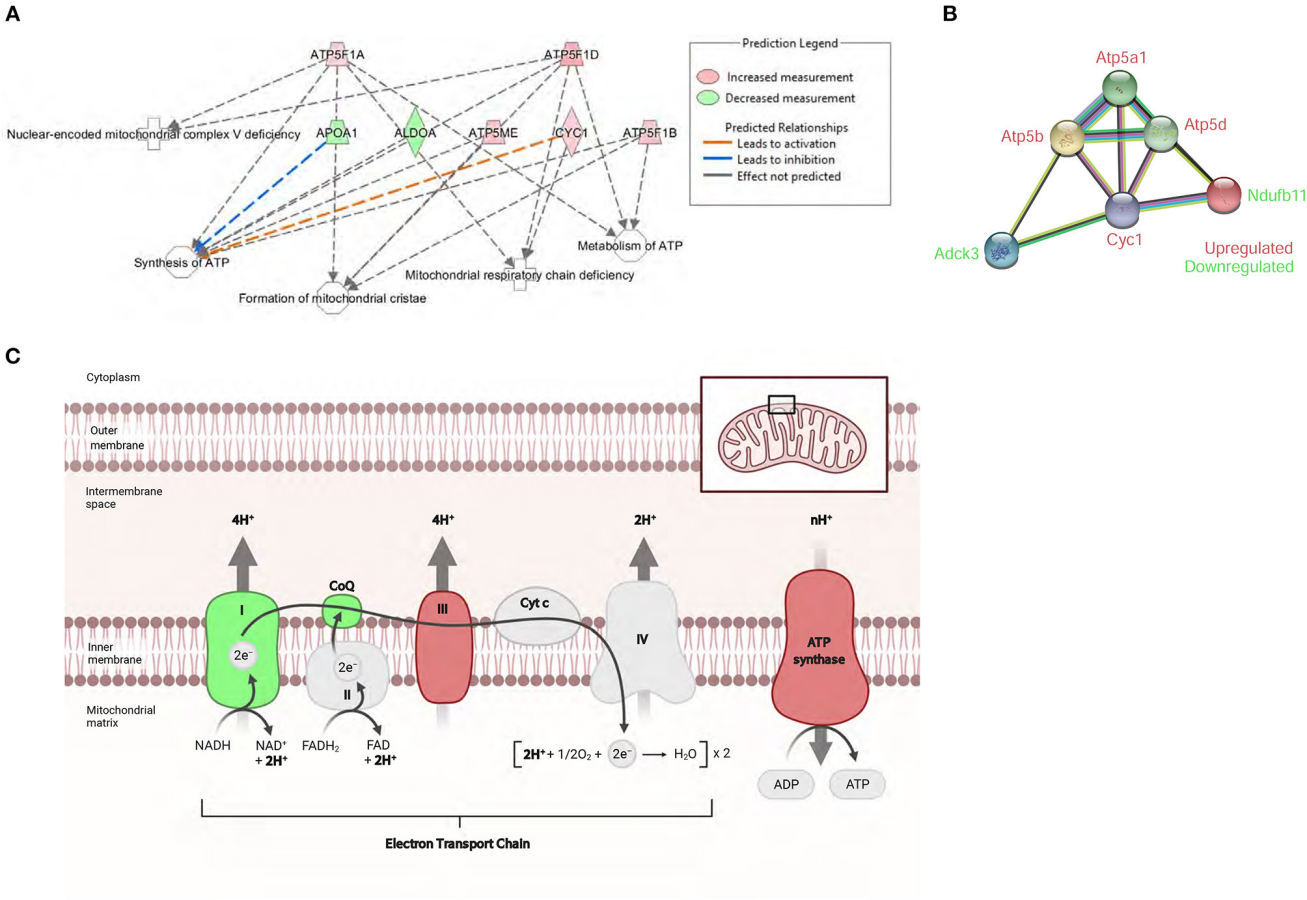
Proteomic profile of *db/db* mice suggests severe mitochondrial metabolic dysregulation. **(A)** The top affected cellular function identified by IPA in *db/db* mice was the ATP synthesis network. **(B)** STRING interaction network of differentially expressed mitochondrial proteins in the left ventricle of *db/db* mice. **(C)** Schematic overview of the mitochondrial respiratory electron transport chain and the differentially expressed mitochondrial proteins associated with diabetic cardiomyopathy. Adapted from “Electron Transport Chain,” by BioRender.com (2021). Retrieved from https://app.Biorender.com/Biorender-templates. Symbol color represents expression value, red indicating an upregulation, and green indicating downregulation in our dataset.

Bioinformatics analysis predicted the mitochondrial dysfunction and oxidative phosphorylation (OXPHOS) to be the highest scoring protein networks impacted in diabetic hearts (Figure 5A). Furthermore, the proteomic analysis also identified the increased peptide levels of alpha, beta, and delta subunits of ATP synthase. Moreover, we also found a marked induction of Cytochrome c1 in *db/db* LVs compared to WT LVs. Conversely, compared to healthy controls, *db/db* mice showed a decrease in peptide levels of NDUFB11 and atypical kinase COQ8A/Adck3 (Figure 5B). These data showed both up and downregulation of key regulators of electron transfer in the electron transport chain (ETC) and mitochondrial ATP production (Figure 5C).

**Figure 6:**
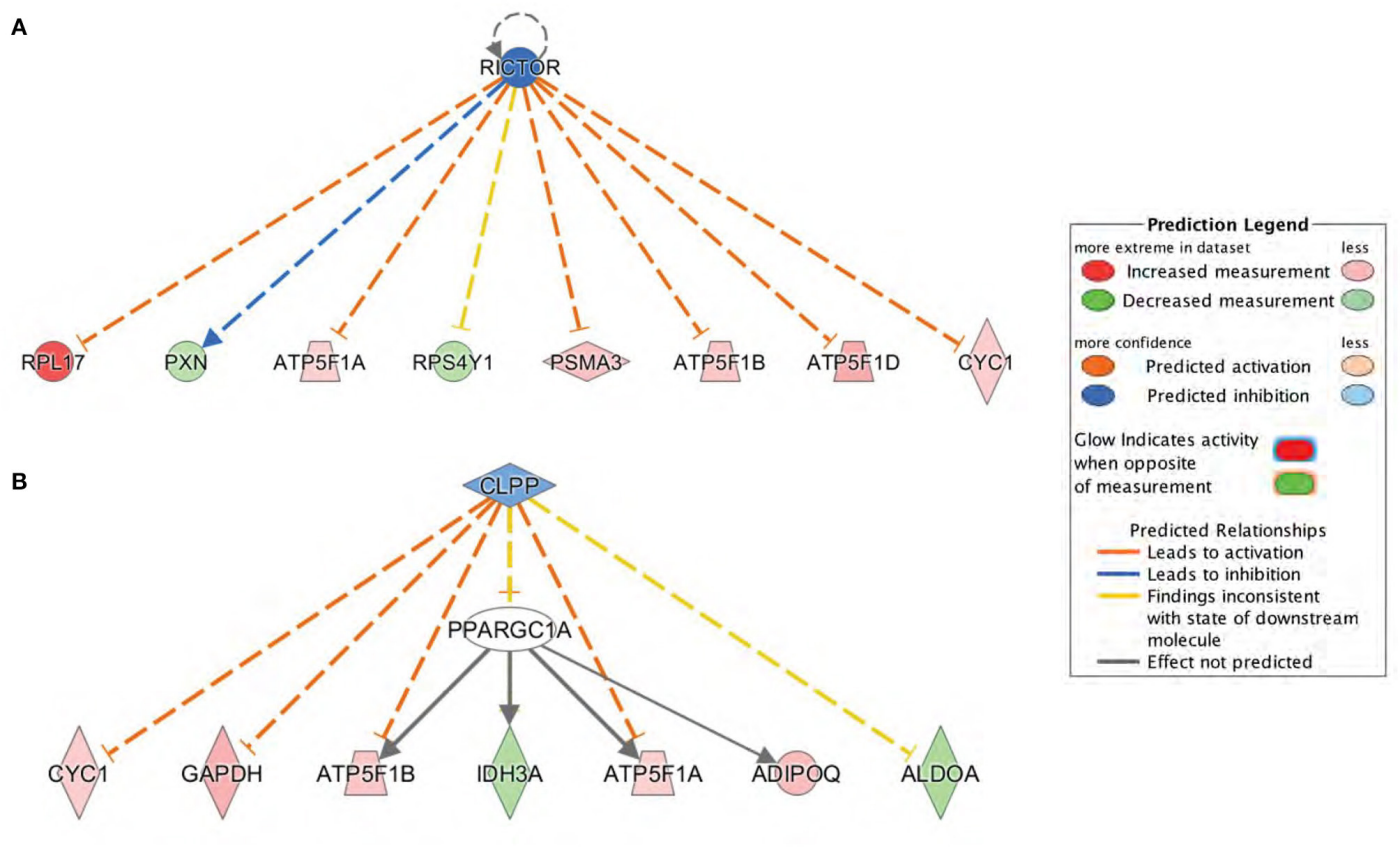
Upstream regulators predicted by Ingenuity Pathway Analysis (IPA) at the proteome level. **(A)** Rapamycin-Insensitive Companion of mTOR (RICTOR) and **(B)** Caseinolytic Peptidase P (CLPP) were the top inhibited upstream regulators predicted in *db/db* mice. Downstream proteins are displayed as networks. Symbol color represents expression value, red indicating an upregulation and blue/green indicating downregulation in our dataset. RICTOR and CLPP modulates the expression or function of downstream proteins listed in figure.

Subsequently, upstream regulator analysis was performed using the IPA program to determine the number of known targets of each transcription regulator present in the *db/db* dataset obtained from the proteomic analysis. Upstream regulator analysis also allowed to compare each differentially expressed protein to the reported relationship in the literature. The top predicted inhibited upstream regulator in the DbCM was Rapamycin-Insensitive Companion of mTOR (RICTOR), a key regulatory subunit that binds to mTOR to form the mTOR Complex 2 (mTORC2) (27). RICTOR leads to inhibition of alpha, beta, and delta subunits of ATP synthase and Cytochrome c1 (28, 29), and its predicted inhibition in our study was associated with the upregulation of the ETC components in the LV of *db/db* mice. RICTOR also leads to activation of Paxillin (PXN) (30), a focal adhesion protein whose inactivation results in a progressive decrease of cardiac contractility and heart failure (31). PXN was decreased in diabetic hearts, validating the predicted inhibition of RICTOR (Figure 6A; Table 2). Another predicted inhibited upstream regulator found in the LV of *db/db* mice was Caseinolytic Peptidase P (CLPP), a mitochondrial matrix ATP-dependent peptidase. CLPP leads to inhibition of alpha and beta subunits of ATP synthase and Cytochrome c1 (32), in addition to Adiponectin (ADIPOQ) and Glyceraldehyde-3-phosphate dehydrogenase (GAPDH). Proteomic analysis identified the upregulation of ATPF1A, ATPF1B, ADIPOQ, and GAPDH in the db/db LVs in our study, validating the predicted inhibition of CLPP (Figure 6B; Table 2).

### Diabetic Cardiomyopathy-Associated Mitochondrial Dysfunction Exhibits Impaired Complex I-Driven Respiration

**Figure 7:**
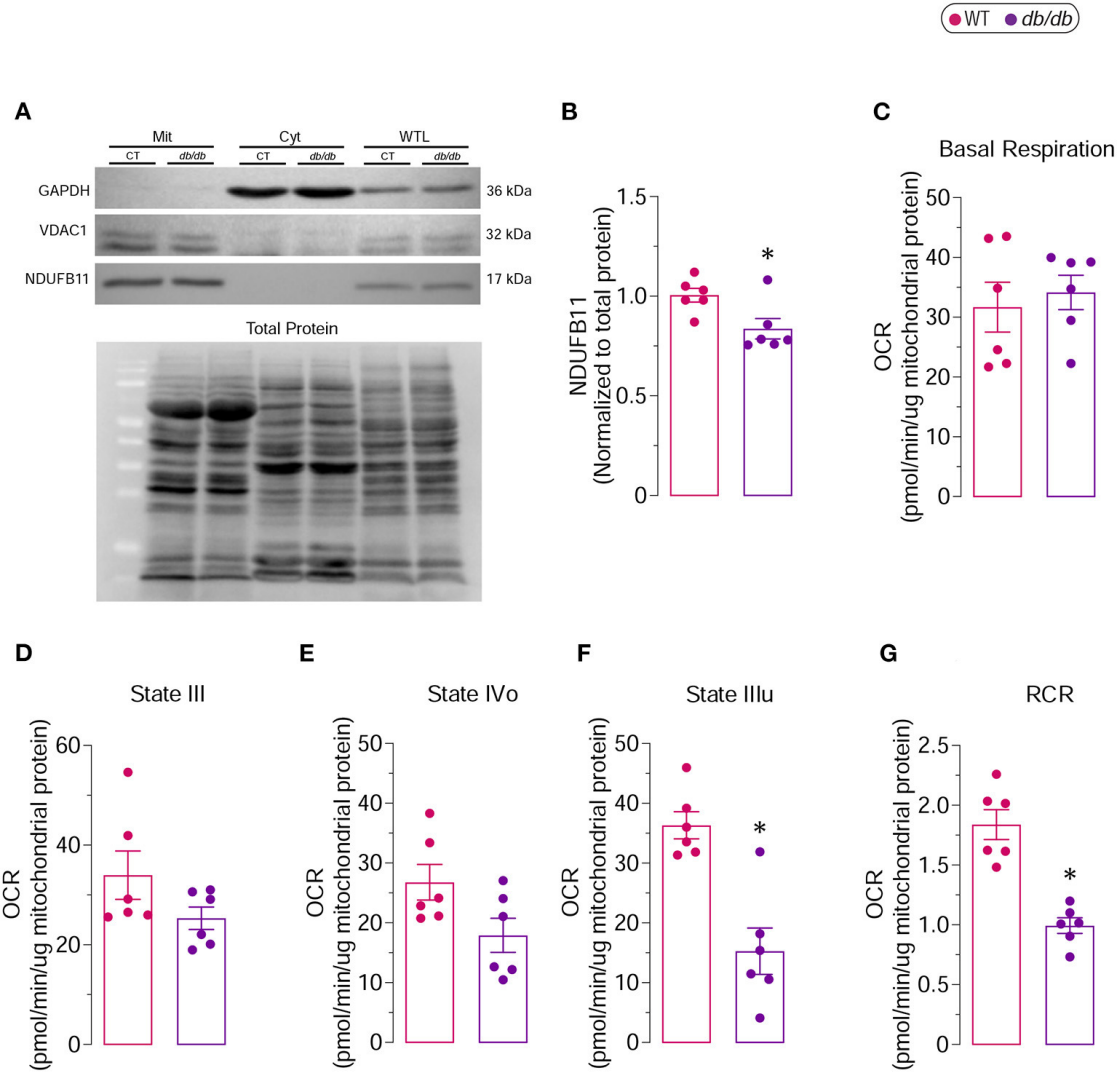
Complex I-driven respiratory capacity in isolated mitochondria from 6 months old *db/db* mice **(A)** Representative immunoblot showing the purity of the mitochondrial (Mit) fraction used for determination of mitochondrial respiratory function. VDAC1 and NDUFB11 were used as mitochondrial markers, while GAPDH was used as cytoplasmic (Cyt) marker. Whole tissue lysate (WTL) protein levels were also evaluated. **(B)** Proteins levels of NDUFB11 normalized to total protein. Oxygen consumption rates (OCR) of isolated mitochondria were measured by Seahorse XF analyzer. **(C)** Basal Respiration, **(D)** State III, **(E)** State IVo, **(F)** State IIIu and **(G)** Respiratory Control Ratio (RCR) are shown. *Represents *p*-value <0.05 between WT (*n* = 6) and *db/db* (*n* = 6) mice.

As our proteomic analysis suggested key regulations of proteins involved in mitochondrial metabolism, we sought to investigate the changes in mitochondrial bioenergetics and performed a mitochondrial coupling assay. Mitochondria were isolated from db/db and WT LVs, and their purity was validated using western blot analysis. The successful mitochondria isolation was corroborated by the presence and absence of mitochondria-specific (VDAC1 and NDUFB11) and non-mitochondrial (GAPDH) proteins in the mitochondrial fraction, respectively (Figure 7A). The proteomic analysis and bioinformatics prediction (Table 2) revealed reduced levels of NDUFB11 in *db/db* mice, a subunit of complex I that facilitates electron transfer to ubiquinone (20). The reduced protein levels of NDUFB11 were also confirmed by the western blot analysis performed on the mitochondrial fraction obtained from LVs of *db/db* mice (Figures 7A,B).

The mitochondrial coupling assay examines the degree of coupling between OXPHOS and ETC; an impaired coupling between OXPHOS and ETC would indicate mitochondrial dysfunction. In the Seahorse extracellular flux analysis, the respiratory chain complex I (RCCI)-driven respiration, which represents the respiration of mitochondria in the presence of substrates but without ADP, did not show any difference in basal respiration between WT and *db/db* groups (Figure 7C). State III, which represents the formation of ATP from ADP and inorganic phosphate, and State IVo, which represents the proton leak due to the inhibition of the ATP synthase by oligomycin, was moderately decreased in mitochondria-derived from *db/db* LVs compared to WT LVs (Figures 7D,E). The state IIIu, an indicator of the maximal respiratory capacity, was significantly decreased in the mitochondria isolated from *db/db* LVs compared to the WT LVs (Figure 7F). The respiratory control ratio (RCR), an index of mitochondrial coupling, which is obtained by dividing the corrected values of State IIIu/State Ivo, was markedly reduced in *db/db* group compared to WT (Figure 7G), indicating increased mitochondrial uncoupling in DbCM. Although the basal respiration remained unchanged, markedly decreased respiratory chain complex I (RCCI)-driven mitochondrial coupling and severely reduced maximal respiratory capacity suggest impaired mitochondrial respiration in *db/db* mice corroborating proteomic discoveries.

## Discussion

DbCM is a complex disorder caused by multifactorial pathology (33). The natural history of DbCM ranges from a short-term physiological adaptation to degenerative changes unable to be repaired by the myocardium, ultimately culminating in an irreversible pathological remodeling (34, 35). Induction of hyperglycemia and hyperlipidemia with progressive accumulation of the respective substrates in cardiomyocytes causes functional and structural changes (36, 37). These gradual changes often begin with diastolic dysfunction, followed by decreased left ventricular systolic function, resulting in HF (3, 38). In the present study, we validated the diabetic phenotype in 6 months old *db/db* mice, which was associated with structural and functional abnormalities of DbCM, including diastolic dysfunction and cardiac hypertrophy. These essential features are related to an established stage of DbCM, validating our experimental model.

HF-related to DbCM is evidently associated with abnormal myocardial energy metabolism with progressive myocardial hypertrophy and fibrosis (4, 39, 40). Despite the increasing number of studies in recent years that attempt to explain the potential pathophysiological mechanisms involved in the genesis of DbCM (21), there is still no clear and comprehensive integration of the pathways involved due to its multifactorial nature. In our research, we used quantitative shotgun proteomics to track the changes in the protein content, and subsequently, the proteomic profile in LVs of type 2 diabetic *db/db* mice. Proteomics analysis provides an unbiased experimental tool for the identification of aberrant protein expressions associated with disease, revealing potential signaling cascades that can be targeted therapeutically. Here we identified the shift in reactome priorities from calcium handling and muscle contraction in healthy mice to energy metabolism and mitochondrial dysfunction in DbCM. Mitochondria play a pivotal role in integrating cellular energy metabolism and cell survival (41). In type 2 DM, the impairment of mitochondrial function leads to a significant ROS production, further contributing to DM-induced myocardial dysfunction (42, 43). Proteomic analysis showed that the peptide levels of alpha, beta, and delta subunits of ATP synthase were upregulated in the LVs of *db/db* mice. These subunits form the F1 domain, a catalytic assembly of the enzyme critically involved in ATP synthesis (44). We also found a marked induction of Cytochrome c1 in LVs of *db/db* mice, which is a catalytic core subunit of the complex III, that catalyzes the transfer of electrons from coenzyme Q to Cytochrome c (45). By means of electron transfer, Cytochrome c1 plays an important role in the elevation of mitochondrial membrane potential, by using its heme group as a redox intermediate to transport electrons between complex III and complex IV (46). Importantly, studies have shown that increased levels of mitochondrial Cytochrome c are early events that precede the onset of apoptosis (47). We hypothesize that increased Cytochrome c1 levels may represent an adaptive mechanism by which diabetic heart attempts to increase electron transfer, and thereby enhance mitochondrial ATP production.

Contrarily, in *db/db* mice, we found decreased peptide levels of NDUFB11, a subunit of complex I that catalyzes the transfer of electrons to ubiquinone, and is considered an important factor in the regulation of mitochondrial respiration (20). Although basal respiration remained unchanged in LVs of *db/db* mice, metabolic assessment exhibited severely reduced rate of state IIIu respiration (maximal respiratory capacity) and decreased respiratory control, during the oxidation of the complex I-linked substrates in isolated mitochondria from *db/db* LVs. Combination of proteomic and metabolic assessments suggest that the restricted proton pumping by complex I may induce a slower and prolonged proton entry into complex V after the addition of ADP, leading to alterations in mitochondrial oxidative capacity and coupling of oxygen consumption, eventually affecting ATP production (48). We also observed a downregulation of atypical kinase COQ8A in the proteomic profile of type 2 diabetic LVs. COQ8A is an essential lipid-soluble electron transporter involved in the biosynthesis of ubiquinone and in the energetic movement of electrons through the ETC (49). Interestingly, DM-associated mitochondrial dysfunction has also been linked with metabolic deficits. The cardiac demand for energy comes predominantly from mitochondrial OXPHOS, which accounts for 95% of total ATP produced. However, in the chronic diabetic state established in 6 months old *db/db* mice, the ability of the heart to switch between available oxidizable substrates is impaired, and in this condition, the heart depends almost exclusively on fatty acid metabolism, which increases mitochondrial damage (50, 51). In our study we found that despite attempts by hearts from the diabetic mice to upregulate some ETC elements, which results in preserved basal mitochondrial metabolism, decreased levels of key components contribute to impaired maximal respiratory capacity, critically affecting ability of the diabetic heart to respond to increased metabolic needs. It is likely that ATP synthase subunits and Cytochrome c1 may also be downregulated with prolonged persistence of the diabetic phenotype. At first, the protective mechanism of mitochondrial function seems to be present, favoring the myocardial redox environment essential for the resting contractile. However, our study suggests that diabetic hearts have altered expression of essential mitochondrial peptides, which may contribute to the impaired mitochondrial bioenergetics contributing to the establishment of DbCM in this model.

In type 2 DM, the accumulation of ectopic lipids in the heart has also been associated with reduced cardiac efficiency. As lipid accumulation and plasma free fatty acid levels increase in type 2 DM, adverse effects of lipid accumulation on cardiac structure and function have been discussed as a potential mechanism for DbCM. *Ex vivo* perfusion of murine hearts from obese mice with free fatty acid demonstrated increased oxygen consumption and reduced ATP-to-Oxygen ratio when compared to glucose perfusion; these changes in the ATP-to-Oxygen ratio were too large to be explained by changes in the substrate metabolism, and were found to be associated with increased mitochondrial uncoupling (52, 53). Moreover, increased fatty acid metabolism in these hearts was associated with increased expression of mitochondrial uncoupling proteins (54). Similarly, reduced mitochondrial oxidative capacity inspite of increased mitochondrial biogenesis were observed in *db/db* hearts (55). Cytosolic as well as mitochondrial lipidic environment has been proposed to play a key role in regulation of mitochondrial metabolism, and remains to be investigated. Integrated proteomic and lipidomic analyses of diabetic heart may provide novel insight into the molecular state and underlying molecular mechanisms of DbCM.

IPA software was used to facilitate the organization and interpretation of the proteomic data in our study, which enabled prediction of upstream regulators to diseases and functions. RICTOR, an obligate regulatory subunit of mTORC2 (27), was predicted to be inhibited in DbCM. Activation of mTORC2 modulates mitochondrial function *via* Akt (28), and regulates cell survival *via* its anti-apoptotic effects in cardiac hypertrophy and myocardial ischemia (56). Inhibition of RICTOR expression has been demonstrated to block mTORC2 assembly and activity (57). RICTOR deletion from cardiomyocytes inactivates mTORC2, but does not modify basal cardiac function and geometry. However, RICTOR-deficient hearts display reduced cardiac performance when challenged by haemodynamic stress, which leads to cardiac dysfunction and dilatation (58). While studies indicate that activating autophagy in cardiomyocytes by inhibiting mTORC1 may prevent the aggravation of DbCM (59, 60), the role of RICTOR/mTORC2 in DbCM remains largely unknown. Additionally, IPA-based bioinformatics analysis also predicted CLPP to be inhibited in the LV of *db/db* mice. CLPP is a mitochondrial peptidase essential for maintaining protein quality control and mitochondrial function. Protease-mediated quality control is the first line of defense against mitochondrial damage and involves the degradation of non-assembled proteins that result from mitonuclear imbalance, and proteins that are damaged or misfolded as result of ROS (61).

Both RICTOR and CLPP are involved in the maintenance of cardiac homeostasis, and alterations in the levels of these regulators may contribute to pathological remodeling and cardiac dysfunction. Cells with genetic deletion of RICTOR exhibit defects in cell polarity and cytoskeletal architecture (62), whereas cardiac-specific knockdown of RICTOR exacerbated cardiac remodeling and dysfunction after myocardial infarction (63). Accordingly, mTORC2 activation has been shown to mediate the cardioprotective effects of hydrogen sulfide in response to ischemia/reperfusion in rats (64). Muscle-specific CLPP deficiency can partially restore mitochondrial protein synthesis, improving mitochondrial respiratory activity and attenuating pathological cardiac remodeling (65). Whereas, CLPP knockout mice exhibit reduced prenatal/postnatal survival, growth retardation, movement impairment, mild respiratory defects, and female and male infertility (65, 66). Indeed, further investigations are highly warranted to better understand the role of RICTOR and CLPP in initiation, progression and establishment of DbCM. In addition to mitochondrial dysfunction, the Integrin-Linked Kinase (ILK) signaling has also been identified as a top canonical pathway through IPA analysis in *db/db* mice LVs. ILK is a broadly expressed serine/threonine-protein kinase that binds to the cytoplasmic tail of β integrins, linking the interactions of cellular matrix to signals that regulate cytoskeletal remodeling and cellular processes such as growth, proliferation, survival, and differentiation (67, 68). As a major regulator of cytoskeletal remodeling, ILK pathway remains of a major interest in various cardiovascular disease.

Growing experimental research has been conducted to better understand the sexual dimorphism in the molecular mechanisms and outcomes of DbCM. However, the sex-specific differences at the level of the myocardium remain largely unknown. A potential limitation of our study is the absence of female subjects, as this could support a better comprehension of the sexual dimorphism in DbCM. Interestingly, Estrogen Receptor Signaling has been identified as a top canonical pathway with predicted activation through IPA analysis in male *db/db* mice LVs. Further research is critical to ascertain the role of estrogen in cardiovascular complications of DM. Furthermore, the validation of our findings in other independent models of DbCM, and explanted human hearts to understand the translational potential of our investigations are paramount. Moreover, although the present study identified mitochondrial dysfunction in diabetic hearts, further studies at various stages of DbCM onset and progression may shed light on “cause-and-effect” relationship between mitochondrial (dys)function and diastolic (dys)function. In summary, the present study found that diabetic LVs displayed altered expression of peptides involved in key mitochondrial metabolic processes together with the coordinated downregulation of cytoskeletal proteins. Further investigations into cause-and-effect relationship may provide novel insight into molecular mechanism of DbCM, with a potential to developing novel therapeutic targets.

## Data Availability Statement

The data presented in the study are deposited in the PRIDE repository, accession number PXD029566.

## Ethics Statement

The animal study was reviewed and approved by the Animal Care Committee, University of Calgary.

## Author Contributions

KG, AJ, and VP designed the research. KG, AJ, LA, NB, PE, RS, DY, DB, JS, and AD acquired and analyzed data. KG and VP wrote the manuscript. VP is the guarantor of this work and, as such, has full access to all the data and takes responsibility for the integrity of data and the accuracy of data analysis, and supervised and managed the funding. All authors critically revised and approved the final version of the manuscript.

## Funding

This work received support from Canadian Institutes of Health Research (CIHR; operating grant to VP) Project Grant (PJT-165857), Libin Cardiovascular Institute, Cumming School of Medicine (Start-up operating fund to VP; postdoctoral scholarships to KG and AJ; graduate scholarship in Women Cardiovascular Health to PE), Alberta Diabetes Institute (operating funds to VP), and Natural Sciences and Engineering Research Council (NSERC; operating grant to AD) Discovery Grant (DGECR-2019-00112).

## Conflict of Interest

The authors declare that the research was conducted in the absence of any commercial or financial relationships that could be construed as a potential conflict of interest.

## Publisher's Note

All claims expressed in this article are solely those of the authors and do not necessarily represent those of their affiliated organizations, or those of the publisher, the editors and the reviewers. Any product that may be evaluated in this article, or claim that may be made by its manufacturer, is not guaranteed or endorsed by the publisher.

## Abbreviations

DM: diabetes mellitus
CVD: cardiovascular disease
HF: heart failure
DbCM: diabetic cardiomyopathy
AGE: advanced glycated end-products
LV: left ventricular
WT: wild-type
*db/db*: membrane-bound leptin receptor deficient mice
FS: fractional shortening
EF: ejection fraction
E: early peak filling
A: late peak filling
IVRT: isovolumic relaxation time
LC-MS/MS: liquid chromatography and tandem mass spectrometry
SAMS: Southern Alberta Mass Spectrometry
Easy-Nlc: nanoflow liquid chromatography
AGC: auto gain control
STRING: search tool for the retrieval of interacting genes
IPA: ingenuity pathway analysis
LVPWd: LV posterior wall thickness at diastole
LVPWs: LV posterior wall thickness at systole
MPI: myocardial performance index
OXPHOS: oxidative phosphorylation system
ETC: electron transport chain
RICTOR: rapamycin-insensitive companion of mTOR
mTORC2: mTOR complex 2
PXN: paxillin
CLPP: caseinolytic peptidase p
ADIPOQ: adiponectin
GAPDH: glyceraldehyde-3-phosphate dehydrogenase
ILK: Integrin-Linked Kinase.
